# Alprostadil associated with low molecular weight heparin to treat limb ischemia caused by SARS-CoV2

**DOI:** 10.1590/1677-5449.200072

**Published:** 2020-11-30

**Authors:** Marcos Antonio Bonacorso Manhanelli, Eliud Garcia Duarte, Jamil Victor de Oliveira Mariuba, Fábio Linardi, José Augusto Costa, Julio Cesar Gali, Winston Bonetti Yoshida, Julio Cesar Gali

**Affiliations:** 1 Pontifícia Universidade Católica de São Paulo – PUC-SP, Sorocaba, SP, Brasil.; 2 Conjunto Hospitalar de Sorocaba, Sorocaba, SP, Brasil.; 3 Hospital Santa Mônica, Cirurgia Vascular e Endovascular, Vila Velha, ES, Brasil.; 4 Universidade Vila Velha – UVV, Vila Velha, ES, Brasil.; 5 Universidade Estadual Paulista “Júlio de Mesquita Filho” – UNESP, Botucatu, SP, Brasil.; 6 JJMED, Sorocaba, SP, Brasil.

**Keywords:** COVID-19, thromboembolism, limb ischemia, arterial thrombosis, heparin, COVID-19, tromboembolismo, isquemia de membros, trombose arterial, heparina

## Abstract

The current coronavirus pandemic has already taken a great toll globally, causing massive morbidity and mortality. One of its severe forms is a thrombophilic state that can damage several systems. This article reports the case of 60-year-old female patient who presented with mild flu symptoms, which turned out to be a SARS-CoV2 infection, and ended up developing arterial thrombosis with limb ischemia in a private care hospital in Sorocaba, São Paulo, Brazil. Considering this progression, we decided to intervene with low molecular weight heparin and Alprostadil, achieving a good clinical outcome. Our description aims to identify key points and clinical signs that offer evidence of the therapeutic window and a treatment option for coagulatory presentations of COVID-19.

## Resumo

A atual pandemia de coronavírus já gerou danos profundos ao redor do mundo, causando grande quantidade de morbidades e mortes. Uma das manifestações das formas graves da doença é o estado trombofílico, que pode provocar danos em vários sistemas. Este artigo relata o caso de uma paciente do sexo feminino, 60 anos de idade, que foi internada em um serviço hospitalar privado com sintomas gripais inespecíficos leves, mas que progrediu com trombose arterial e isquemia de membros causada pelo SARS-CoV2. Devido à essa evolução, foi optada pela administração concomitante de heparina de baixo peso molecular e Alprostadil, com bom desfecho clínico. Nossa descrição objetiva identificar pontos-chave e sinais clínicos que evidenciem essa janela terapêutica, bem como uma opção de tratamento para as apresentações coagulatórias da COVID-19.

## INTRODUCTION

From December 2019, when it originated in Wuhan, China, up until July 21, 2020, Coronavirus disease 2019 (COVID-19), the condition caused by the severe acute respiratory syndrome coronavirus 2 (SARS-CoV-2), had already contaminated over 14,700,000 people worldwide and killed more than 600,000.

Among its several presentations, COVID-19 may induce a hyperinflammatory state, which is usually associated with severe cases and statistically significant differences in markers such as C-reactive protein (CRP), interleukin-6 (IL-6), lactic dehydrogenase (LDH), and serum ferritin.^1^

This hyperimmune state contributes to hemostatic anomalies, including abnormal coagulation, with increased prevalence of thrombotic events.^2^ Severe cases may also be at higher risk of vascular injuries due to common intensive care unit (ICU) factors like sedation, immobilization, and use of vasopressors or central venous catheters.

There have been reports of this blood dyscrasia causing acro-ischemia, usually characterized by red to violet macules, plaques, and nodules (chilblain-like lesions), or rounded erythematous macules and vesicles (erythema multiforme-like lesions).^3^ These lesions may rapidly progress to cyanosis, skin bullae, and gangrene.

D-dimer and prothrombin time (PT) are two of the most prominent factors that can be monitored and constitute evidence of hemostatic abnormalities in COVID-19.^4^ These laboratory tests are also related to worse outcomes.^4^ Nonetheless, there is still no consensus on cutoff values.

The objectives of this paper are to explain why the treatment administered was chosen and discuss further treatment possibilities.

## CASE DESCRIPTION

A 60-year-old female patient was admitted to a private hospital complaining of fever, myalgia, headache, and malaise lasting five days. She has insulin-dependent type 2 diabetes mellitus and had attended a crowded event in São Paulo ten days prior to seeking care.

She also reported nausea and dyspnea and, after ground glass opacity findings on computed tomography (CT) of the chest, she was hospitalized with isolation because of suspected COVID-19.

Treatment began with Ceftarolin fosamil 1200 mg/day, Azithromycin 500 mg/day, Oseltamivir 75 mg/day, and Hydroxychloroquine 600 mg/day. Day 1 laboratory test results were as follows: D-dimer 0.3 ug/mL, LDH 315 U/L, blood urea 37 mg/dL, creatinine 1.0 mg/dL, and neutrophil count 4630 U/L (Table 1 – reference ranges).

**Table 1:** (test results by days since admission, showing key events and reference ranges).

**EXAM**	**Mar-21 (DAY 1)**	**Mar-24 (DAY 4)** *	**Mar-25 (DAY 5)**	**Mar-29 (DAY 9)**	**Mar-30 (DAY 10)**	**Mar-31 (DAY 11)**†	**Apr-1 (DAY 12)**	**Apr-3 (DAY 14)**‡	**Apr-4 (DAY 15)**	**Apr-13 (DAY 24)**	**Apr-18 (DAY 29)**§
**C-REACTIVE PROTEIN**		15.1	26.6	4.8	2.5	13.5	27.2	15.5	17.1	11.8	16.9
**D-DIMER**	0.3		0.7	2.7				0.8		2.6	1.4
**BLOOD UREA**	37		23	63	82	90	96	81	78	74	42
**CREATININE**	1		1.1	1.4	1.3	1.2	1.2	1.2	1.2	0.8	0.6
**CREATINE PHOSPHOKINASE**			60	327		2757	3329	2767	1901	170	43
**INR (international normalized ratio)**			1.06	0.96	0.98	1.03	1.21	1.09	1.07	1.13	1.17
**PROTHROMBIN TIME**			13.8s	12.8s	13.1s	13.5s	15.3s	14.2s	13.9s	14.6s	15.0s
**APTT (activated partial thromboplastin time)**			38.3s	36.5s	30.6s	45.8s	53.6s	53.6s	50.6s	31.1s	49.0s
**APTT pool ratio**			1.10	1.05	0.88	1.32	1.54	1.54	1.45	0.93	1.46
**LACTIC DEHYDROGENASE**	315		618	375			412	341	338		243
**FERRITIN**				420			580				628
**PLATELET COUNT**	118K	130K	197K	217K	186K	228K	227K	267K	331K	298K	316K
**ERYTHROCYTE COUNT**	4.8	4.4	4.53	4.04	3.77	4.25	3.78	3.28	3.28	3.23	3.03
**HEMOGLOBIN**	12.5	11.4	11.8	10.5	9.7	11	9.6	8.5	8.5	8.5	8.2
**HEMATOCRIT**	38.2	35.2	38.3	34.8	31.7	34.8	31.2	27.3	27.2	27	24.5
**LEUCOCYTE COUNT**	5910	4520	14380	10840	9450	14650	11590	10520	10850	11880	11830
**NEUTROPHIL COUNT**	4630	3313	12511	9843	8316	12409	9817	9005	9765	8910	9346
**LYMPHOCYTE COUNT**	1010	868	288	336	473	894	707	1084	326	832	1183
**EOSINOPHIL COUNT**	0	9	0	0	0	147	0	105	109	950	473
**OXYGEN SATURATION (%)**	96.30%	94.70%	99.60%	97.10%	95.20%	91.90%	96.80%	97.00%	95.60%	93.20%	96.80%
**BLOOD pH**	7.397	7.461	7.194	7.267	7.561	7.535	7.560	7.479	7.475	7.521	7.474
											
**TEST**	REFERENCE NORMAL RANGE										
C-Reactive Protein	< 0.5 mg/dL										
D-dimer	< 0.5 ug/mL										
Blood Urea	16.6-48.5 mg/dL										
Creatinine	0.5-0.9 mg/dL										
Creatine Phosphokinase	26-192 U/L										
INR (international normalized ratio)	0.8-1.1										
Prothrombin time	11-15 seconds										
APTT	30-50 seconds										
APTT pool ratio	< 1.26										
Lactic Dehydrogenase	133-225 U/L										
Ferritin	13-150 ng/mL										
Hemoglobin	10.90-17.50 g/dL										
Hematocrit	34-52%										
Leucocyte count	4000-10000 /uL										
Neutrophil count	1800-7200 /uL										
Lymphocyte count	800-3500 /uL										
Eosinophil count	40-400 /uL										
Oxygen Saturation (%)	> 96%										
Blood pH	7.370-7.450										

* intubation, †LMWH full dosage, ‡dilated pupils; §ICU discharge.

On day 4 the patient’s dyspnea and radiological patterns worsened. Her clinical condition deteriorated, arterial blood gas analysis revealed hypoxia, with 94.7% oxygen saturation, and orotracheal intubation was needed with mechanical ventilation and sedation. Laboratory data showed a neutrophil spike (12511 U/L) along with an important C-Reactive Protein (CRP) elevation (from 15.1 mg/dL to 26.6 mg/dL).

Oxygen saturation improved, but blood pressure began oscillating excessively, requiring catheter insertion via puncture without dissection of the upper right radial artery to continuously monitor mean blood pressure. However, patency through this arterial access was lost. It was then decided to switch to the lower left limb dorsalis pedis artery, but puncture resulted in a similar occlusive failure. However, it is very important to emphasize that the aforementioned vessels had not shown signs of major dyscrasia before being pierced.

On day 9, D-dimer was 2.7 ug/mL, with sustained neutrophilia, lymphopenia, elevated blood urea and creatinine (63 mg/dL and 1.4 mg/dL, respectively), and creatine phosphokinase (CPK) had increased to 2757 U/L. However, CRP had decreased significantly, to 4.8 mg/dL (Table 1).

The patient then developed fixed cyanosis simultaneously in all right hand fingers and the palmar region, accompanied by extensive necrotic bullae compromising most of the forearm, on day 10. Hand temperature was preserved, with full ulnar pulse but pronounced livedo reticularis was observed extending from the axilla to the forearm lesions. The contralateral hand had no remarkable findings.

There was also fixed cyanosis in all toes of the left foot and the plantar midfoot area, with necrotic bullae involving the dorsal area of the foot, up to the ankle. Foot temperature was preserved, with full posterior tibial pulse but no dorsalis pedis pulse. A duplex ultrasound scan of the anterior tibial artery was performed, which presented occlusion of the distal third and plantar arch compromise. Livedo reticularis was observed up to the knee and thigh, while the contralateral limb had no stigmata of occlusive peripheral arterial disease. Ultrasound scan also revealed triphasic flow in the posterior tibial and fibular arteries of the left limb.

From days 10 to 11, she had spikes in CRP, urea, and neutrophil count, with progressive clinical deterioration. We interpreted this as evidence that the patient was in a thrombophilic state, with microvasculature implications. On day 11, we opted to intervene with full therapeutic dosage heparinization, 1mg/kg low weight molecular heparin, every 12 hours, and Alprostadil 40 mcg on the same schedule, in order to try to improve microvasculature, while recommending her limbs and extremities were kept warm.

**Figure 1:**
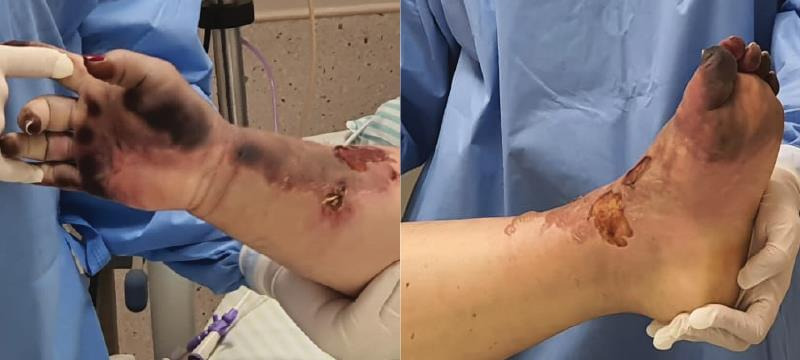
April 3rd, day 14, necrotic areas of right hand and left foot.

On day 14 (Figure 1), the patient showed significant improvement in livedo reticularis, coinciding with a D-dimer regression to 0.8 ug/mL and a CPK regression to 2767 U/L. Four days later, the areas of cyanosis had become delimited and limb reperfusion was progressing, while bullae had progressed to hardened eschars, with granulation tissue beneath them. D-dimer and CPK were still decreasing, to 0.4 mg/dL and 669 U/L, respectively. At this point, the patient’s pupils were dilated bilaterally and unresponsive to light.

**Figure 2:**
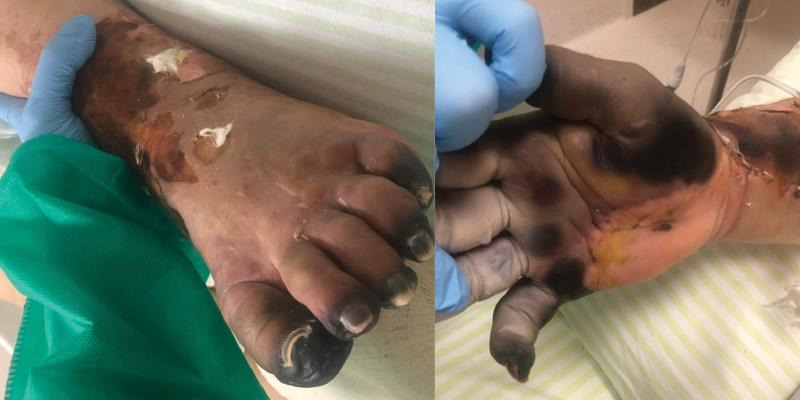
April 13th, day 24, necrotic areas of right hand and left foot.

Twelve days after coagulation therapy began, on day 24 (Figure 2), the patient was awake and aware of time and place. She had no sensibility or mobility in the fingers of her right hand, with superficial palm necrosis. Hand temperature was preserved, with full ulnar pulse and the viable tissue area was restricted to 66% of the hand. In the left foot, the limit of viability was just above the toes.

**Figure 3:**
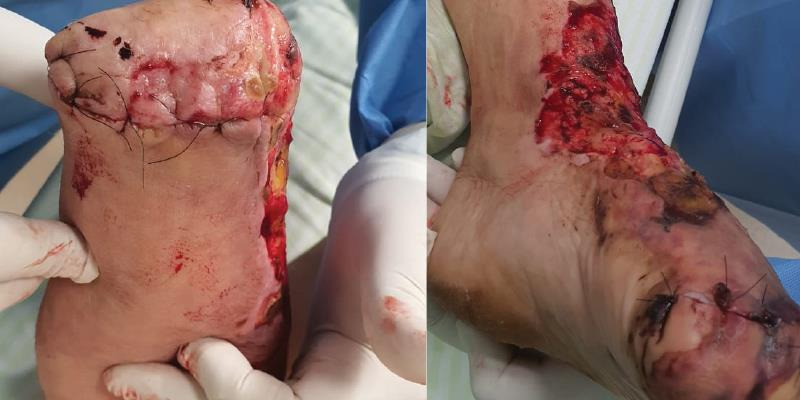
April 20th, day 31, right hand and left foot amputations, 4th postoperative day.

**Figure 4:**
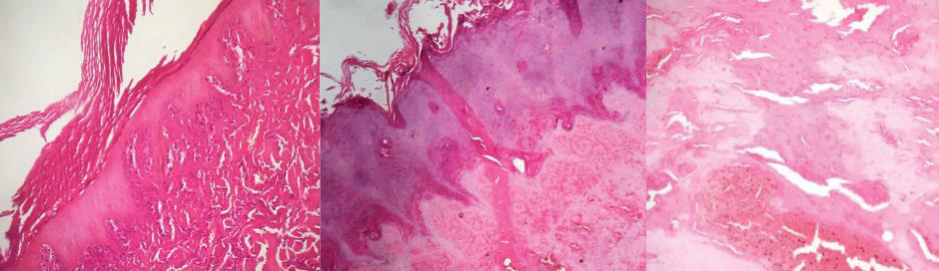
Microscope slides, showing edema, coagulation necrosis, and inflammatory process.

**Figure 5:**
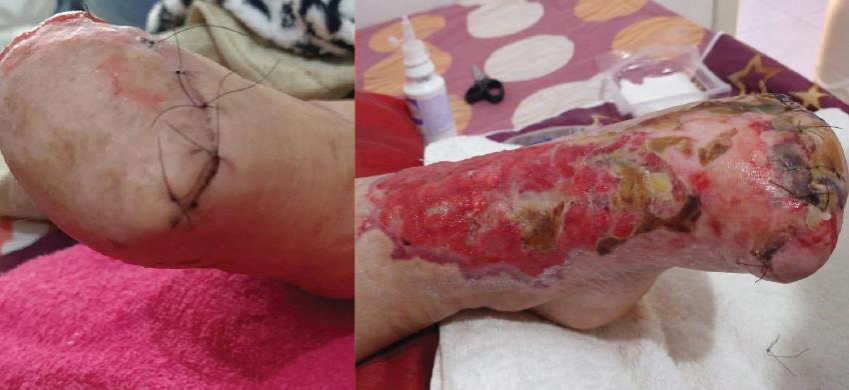
May 7th, day 48, right hand and left foot amputations, 21st postoperative day.

On day 26, amputations were performed at the distal forefoot and wrist (Figures 3, 4). The decision to amputate at the wrist was taken after discussion with hand surgery specialists, to facilitate planning of prosthetics. The patient was discharged from the ICU on day 29, with 1.4 ug/mL D-dimer, 42 mg/dL blood urea, and 44 U/L CPK. On day 48, the patient was clinically stable with improvement in the extremities (Figure 5).

## DISCUSSION

Not much is currently known about COVID-19, but part of its pathogenesis is directly linked to angiotensin-converting enzyme 2 (ACE2)^5^ and its expression in target organs,^6^ while another component is rooted in the host immune response.^7^^,^^8^

Our report does not differ from current acknowledged data on SARS-CoV2 infections, considering the noticeable patterns in the subject’s test results, with major increases in neutrophil count, ferritin, CRP and LDH (Table 1). It is also critical to state that there were no signs that the patient’s clinical decline was caused by any of the medications administered, including hydroxychloroquine or azithromycin.

We also used a well-accepted drug in COVID-19 treatment, low molecular weight heparin (LMWH),^9^^,^^10^ because disseminated intravascular coagulation (DIC) patterns are confirmed as a factor indicative of poor prognosis for disease outcome^4^ and adequate treatment of this condition may be beneficial.

Besides anti coagulation and anti-viral effects, the good results may be partly due to anti-inflammatory activity.^11^ However, the therapeutic window for this drug class is not yet clear. Observing our patient’s clinical oxygen saturation decline and other cases of silent hypoxia, it is imperative to ponder the underlying cause of hypoxemia and tachypnea, i.e. whether they are induced by respiratory failure alone or by a hypercoagulability state that compromises both pulmonary surface exchanges and peripheral blood flow.

One fact that might support the latter hypothesis is that our patient was awake and communicating with a low oxygen saturation (Table 1) and her oxygen saturation kept falling on mechanic ventilation, despite oxygen availability.

There is a possibility that the problem is not in the airways and diaphragm and so unnecessary early intubation may injure otherwise healthy lungs because of the pressure,^12^ disrupting tissue integrity, leading to neutrophil extravasation and cytokine release and resulting in worse outcomes. However, LMWH may also be an adjuvant in this scenario, considering the mechanism of its protective role in reducing ventilation-induced lung injury.^13^

It is therefore possible that the therapeutic window for anticoagulants in COVID-19 may actually open sooner than current recommendations indicate. Skin lesions such as the ones described by Fernandez-Nieto et al.,^3^ along with hypoxia, may be enough to indicate preemptive anticoagulation.

Furthermore, Alprostadil might have been an additional element in the patient’s improvement. It is the naturally occurring prostaglandin E1 (PGE1), a potent vasodilator agent with several pharmacologic actions, including increased peripheral blood flow and inhibition of platelet aggregation.^14^

In rats, Alprostadil had a positive effect on acute respiratory distress syndrome (ARDS), possibly through suppressing the nuclear factor kappa-light-chain-enhancer of activated B cells (NF-κB) signaling pathway and decreasing ACE expression,^15^ both important mechanisms in COVID-19.

IL-6 and tumor necrosis factor alpha (TNF-α) may indirectly interfere with PGE1 synthesis by affecting fatty acid metabolism,^16^ and their availability is also influenced by heparin, considering its close association with lipoprotein lipase (LPL).^17^

Prostaglandins may be a good option when dealing with acute lung injuries (ALI), by acting like nitric oxide, but safer.^18^ Furthermore, infections can cause a range of dyscratic blood responses, not only by disrupting tissue integrity,^19^ but also by interfering directly or indirectly with coagulation mediators.^20^^-^22

We can also point out that coronavirus may summarily manifest in three forms: asymptomatic^23^; hyper-infection, the classic infection pathway, when the pathogen is the main culprit of damage in target organs; and as an infectious-inflammatory syndrome with immune and dyscratic blood components, where most injuries are caused by the host’s response itself.^24^^,^^25^

Mann et al.^26^ performed a study with 1000 subjects where intra-arterial pressure monitoring through puncture resulted in just two occlusive events, both of which were resolved after cannula removal. Here, thrombosis occurred in vessels where tissue integrity was compromised, taking into account that arterial occlusion occurred in punctured arteries and remained after cannula removal. As described by Iba and Levy,^27^ glycocalyx integrity is paramount in maintaining microcirculatory function during infections. Therefore, the vessel punctures in conjunction with the sepsis-like physiology of COVID-19 **were possibly the causes** of our patient’s clinical deterioration. Thus, rushed invasive interventions may need to be thoroughly reevaluated, since we cannot determine how damaging this jeopardization of endothelial cohesion was.

We conclude that more trials are required to pinpoint the therapeutic window and to further recommend LMWH and Alprostadil as treatment for COVID-19 related disorders, especially because this is the first literature report, to date, in which Alprostadil was used. Moreover, endothelial dysfunctions are only part of a larger SARS-CoV2 picture; therefore, each patient’s context should be evaluated from a wider perspective.
